# 236. Treatment of Recurrent *Clostridioides difficile* Infection With RBX2660 in Patients ≥ 65 Years Old With Underlying Comorbidities

**DOI:** 10.1093/ofid/ofac492.314

**Published:** 2022-12-15

**Authors:** Glenn S Tillotson, Paul Feuerstadt, Laurie Archbald-Pannone, Laurie Archbald-Pannone, Stuart Johnson, Samson Ng, Masakazu Ando, Adam Harvey

**Affiliations:** GST Micro LLC, NORTH, Virginia; Yale University School of Medicine/PACT-Gastroenterology Center, Westport, Connecticut; University of Virginia, Charlottesville, Virginia; University of Virginia, Charlottesville, Virginia; Hines VA Hospital and Loyola University Medical Center, Hines, Illinois; Ferring Pharmaceuticals, Parsippany, New Jersey; Ferring Pharmaceuticals, Parsippany, New Jersey; Ferring Pharmaceuticals, Parsippany, New Jersey

## Abstract

**Background:**

Disruptions to gut microbiota composition can result in dysbiosis and subsequent intestinal colonization by opportunistic pathogens such as *Clostridioides difficile.*^1,2^ The incidence of *Clostridioides difficile* infection (CDI) in persons ≥ 65 years old is greater than in those < 65 years old,^3^ with 1 in 11 CDI patients ≥ 65 years old dying within 1 month of diagnosis.^4^ We report the efficacy and safety of RBX2660, a microbiota-based live biotherapeutic, in patients with recurrent CDI (rCDI) who were ≥ 65 years old with comorbidities. This is a subgroup analysis of the PUNCH CD3 trial (NCT03244644), a prospective, multicenter, randomized, double-blind, placebo-controlled phase 3 trial.

**Methods:**

Participants enrolled in PUNCH CD3 were ≥ 18 years old with documented rCDI who completed standard-of-care antibiotic therapy prior to treatment with RBX2660 or placebo. Treatment success was defined as remaining recurrence-free 8 weeks after intervention. In this subgroup analysis, we assessed outcomes of participants ≥ 65 years old with underlying cardiac disorders, chronic kidney disease (CKD), and gastrointestinal (GI) disorders. The treatment-emergent adverse events (TEAEs) were summarized for the double-blind treatment period within 8 weeks and censored if a patient received open-label RBX2660 after CDI recurrence.

**Results:**

In the modified intent-to-treat population, 119 of 262 participants (45%) were ≥ 65 years old. Of these 119 participants, 42% had a cardiac disorder, 19% had CKD, and 61% had a GI disorder; the respective RBX2660 treatment success rates were 69%, 68%, and 67% (**Figure 1**). In the total safety population, the overall incidence of TEAEs was 52% with RBX2660 treatment compared to 44% with placebo treatment; mild events accounted for most of the difference (40% vs 30%) (**Table 1**). The overall incidence of TEAEs was 51% in RBX2660-treated participants ≥ 65 years old and 61%, 68%, and 51% in those participants with a cardiac disorder, CKD, or GI disorder, respectively. Most TEAEs were mild or moderate in severity and related to a pre-existing condition.

**Figure:**
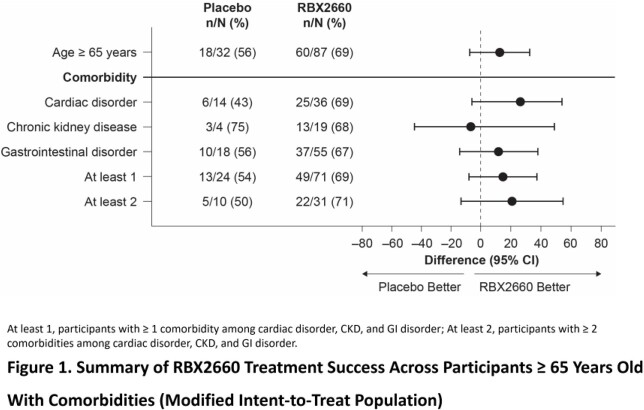

**Figure:**
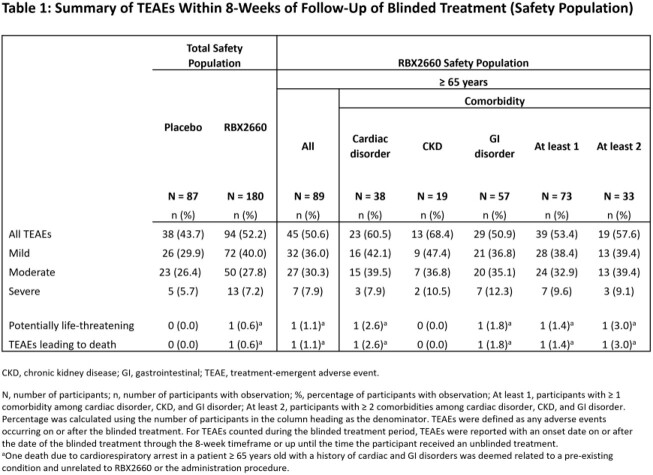

**Conclusion:**

RBX2660 is safe and efficacious across a range of medically complex patients and consistently reduced rCDI in adults ≥ 65 years old, regardless of baseline comorbidities.

**Disclosures:**

**Glenn S. Tillotson, PhD**, Ferring Pharmaceuticals: Advisor/Consultant|Paratek Pharmaceuticals: Grant/Research Support|Spero Pharmaceuticals: Advisor/Consultant|Taro Pharmaceuticals: Advisor/Consultant **Paul Feuerstadt, MD, FACG, AGAF**, Ferring/Rebiotix Pharmaceuticals: Advisor/Consultant|Ferring/Rebiotix Pharmaceuticals: Grant/Research Support|Merck and Co: Advisor/Consultant|SERES Therapeutics: Advisor/Consultant|SERES Therapeutics: Grant/Research Support|Takeda Pharmaceuticals: Advisor/Consultant **Stuart Johnson, M.D.**, Ferring Pharmaceuticals: Membership on Ferring Publication Steering Committee|Ferring Pharmaceuticals: Employee|Summit Plc: Advisor/Consultant **Adam Harvey, PhD**, Ferring Pharmaceuticals: Employment.

